# A Comparison of the Edge Testing of Indirect Composite Layered Zirconia Crowns and Monolithic Zirconia Crowns Without Aging: An In Vitro Study

**DOI:** 10.7759/cureus.62080

**Published:** 2024-06-10

**Authors:** Maaz Vohra, Nabeel Ahmed, Varun Keskar, Amrutha Shenoy

**Affiliations:** 1 Department of Prosthodontics, Saveetha Dental College and Hospitals, Saveetha Institute of Medical and Technical Sciences, Saveetha University, Chennai, IND

**Keywords:** universal testing machine, vickers test, edge testing, indirect composites, monolithic zirconia

## Abstract

Introduction

The main reason veneered zirconia restorations fail is due to porcelain veneer chipping. This chipping usually starts from wear marks on the chewing surface. As a result, small cracks under the contact area can grow into larger ones across the veneer layer. The veneer ceramic layer is more vulnerable to fractures because it has lower toughness and slightly lower stiffness compared to the base framework material. Thus, even when there's significant chipping, the main framework material usually stays protected with a thin layer of veneer ceramic on top. The aim of this in vitro study is to compare the edge strength of Monolithic Zirconia Crowns with that of Indirect Composite Layered Zirconia Crowns without aging.

Materials and methods

This research involved creating 12 hand-layered all-ceramic crowns and 12 indirect composite layered zirconia crowns. The sample size was determined using a G*Power calculation (Heinrich-Heine-Universität Düsseldorf, Düsseldorf, Germany). The zirconia frameworks (Upcera HT White; UPCERA Dental America Inc., Cerritos, CA, US) were milled and sintered following the manufacturer's instructions. For the all-ceramic group, veneering porcelain (e.max Ceram; Ivoclar Vivadent, Schaan, Liechtenstein) was hand-applied. In contrast, the indirect composite group utilized Ceramage (Shofu, Kyoto, Japan). An Instron 4501 universal testing machine (Instron Corp., Canton, MA, USA) was employed for the edge chipping tests, and a Vickers indenter (Shanghai Toyo Diamond Tools Co., LTD, Shanghai, China) was used to apply the load. The mean value for edge chipping was analyzed using an unpaired t-test with IBM SPSS Statistics for Windows, Version 26 (Released 2019; IBM Corp., Armonk, NY, USA). The normality of the data was confirmed, and statistical significance was set at 0.05.

Results

Monolithic Zirconia Crowns (Group 1) require significantly more force (mean: 405 N) to induce an edge chip compared to Indirect Composite Layered Zirconia Crowns (Group 2) (mean: 300 N). The 95% confidence interval (83.43261 N to 109.90072 N) confirms the statistical significance of this difference.

Conclusion

In conclusion, when evaluating restorative materials based on both esthetic and functional criteria, monolithic zirconia stands out due to its combination of strength, esthetic potential, biocompatibility, and versatility.

## Introduction

In the late 1980s, the National Physical Laboratory in London developed the edge chipping test to study hard metal cutting tools. This approach involves generating chips by pressing an indenter near the material's edge. The required force, labeled as F, for chip formation increases as the distance from the edge, known as d, grows. Quinn played a pivotal role in modifying this method for examining dental restorative materials and continued her contributions until her sad demise in 2008. Following her work, research in this area continued and led to the release of three publications in 2014 [1]. Several research groups have utilized this technique to evaluate not just human teeth and dental restorative materials but also a wider range of structural ceramics. Research centered on the longevity of clinical treatments has pinpointed chipping as a leading cause of restoration failures. Although some situations permit the repair of such restorations, there are cases where complete replacement becomes essential. Costa curated eight clinical studies, of which six emphasized chipping in the veneer as a major concern. This specific chipping issue is notably identified as a challenge in modern zirconia restorations [2,3]. Furthermore, there are situations where chipping can occur at the crown's edge during its manufacturing phase, potentially undermining the restoration's overall structural strength [4].

Major fractures originate from initial chips at the edges, leading crowns to split due to the effects of hoop stresses [5]. Unfortunately, many clinical studies often overlook specifics about the size and characteristics of these chipping events [6]. The primary reason for issues with veneered zirconia restorations is the chipping of the top porcelain layer. This type of chipping, frequently initiated by wear marks on the chewing surface, is a specific type of damage from contact. Such damage causes tiny cracks to develop beneath the contact point, eventually forming a significant crack across the entire veneer layer [7]. The chipping phenomenon usually affects only the veneering ceramic layer, mainly due to its lower fracture toughness, and to a lesser extent, its lesser modulus compared to the underlying framework material. Thus, in cases of significant chipping, the main core material typically remains protected, frequently preserving a thin layer of ceramic veneer on its surface [8]. The link between damage from contact and persistent thermal stresses has been emphasized as a key factor leading to the frequent chipping observed in clinical scenarios. These residual thermal stresses appear in the veneer material as it cools down, with variables such as cooling speed, the materials' thermal expansion coefficients, and the core-to-veneer thickness ratio playing crucial roles in determining this phenomenon [9]. Rapid cooling after the last firing step is recognized for creating significant tensile stresses in the veneer, which promotes crack development. To address this particular issue of residual stresses during cooling, alternative materials like veneering composites and monolithic zirconia have been developed and improved over the years [10].

In this in vitro study, the authors seek to evaluate the edge durability between Monolithic Zirconia Crowns and zirconia frameworks with indirect composite veneers. The objective is to gain a better understanding of these restorative materials, aiming for more enduring prosthetic solutions with fewer issues in real-world clinical situations. The null hypothesis posits that there's no distinction in the edge-chipping tendencies between the all-ceramic Monolithic Zirconia Crowns and the Indirect Composite Layered Zirconia Crowns.

## Materials and methods

**Figure 1:**
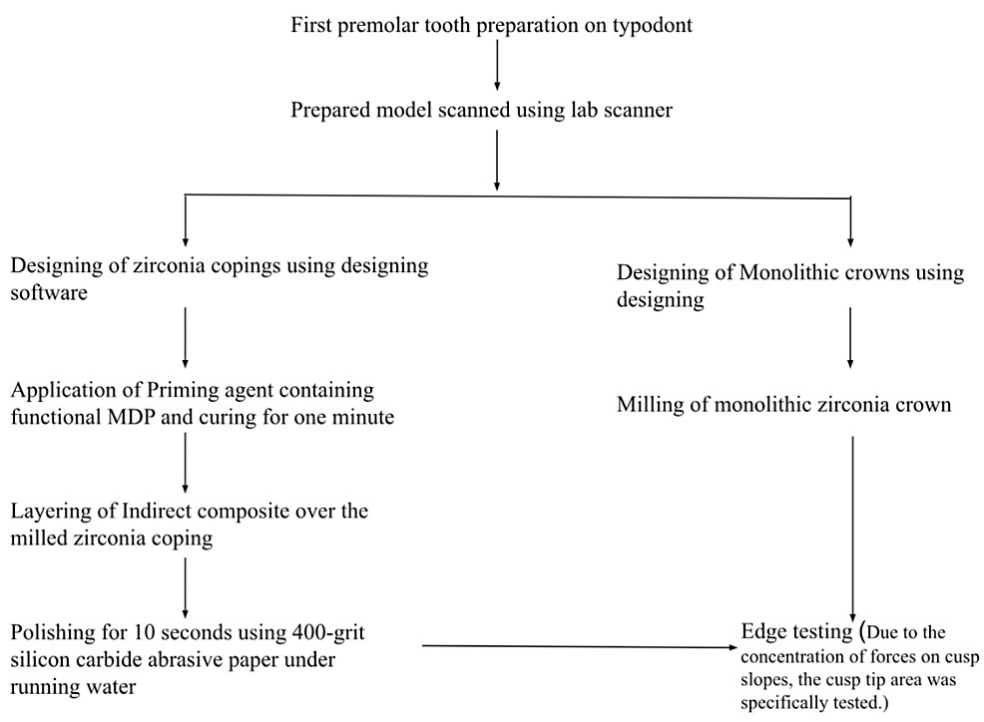
Workflow of the methodology

This in vitro study was designed to have a sample size of 24 (12 in each group named Monolithic Zirconia Crowns and Indirect Composite Layered Zirconia Crowns). This was done based on a previously done study using computerized software: G*Power analysis (Heinrich-Heine-Universität Düsseldorf, Düsseldorf, Germany; Version 3.1) with a significance level of 0.05 and keeping the power at 0.85 and (1-α) was determined as 0.15 [5]. The scientific review board approval number was SRB/SDC/PROSTHO-2105/22/168. Figure 1 depicts the workflow of the methodology.

**Figure 2:**
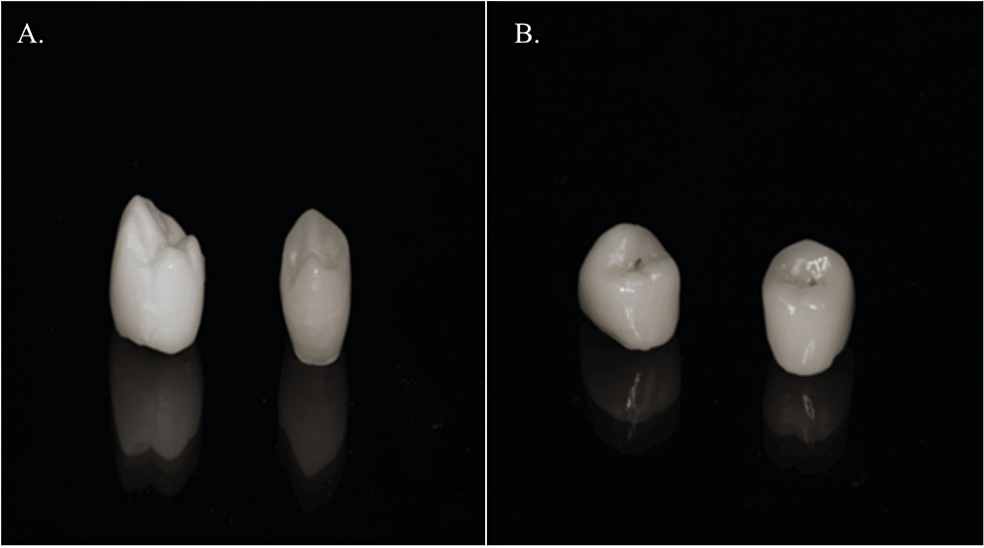
A) Indirect composite layered on zirconia core; B) Monolithic zirconia crown

A single first premolar tooth preparation was done on a Nissin typodont (Nissin Dental Products Inc., Kyoto, Japan). The typodont model was then scanned using a 3shape E series lab scanner (3shape, Copenhagen, Denmark). After obtaining the stereolithography (STL) file from the scan, the design for the coping was created using the 3shape Dental Designer 2021 software. After that indirect composite was layered (Ceramage; Shofu, Kyoto, Japan). Additionally, a design for the monolithic crown was made using the same software. The monolithic crown was fabricated using an Ivoclar ZirCAD prime blank (Ivoclar Vivadent, Schaan, Liechtenstein) (Figures 2A-2B).

Edge testing

**Figure 3:**
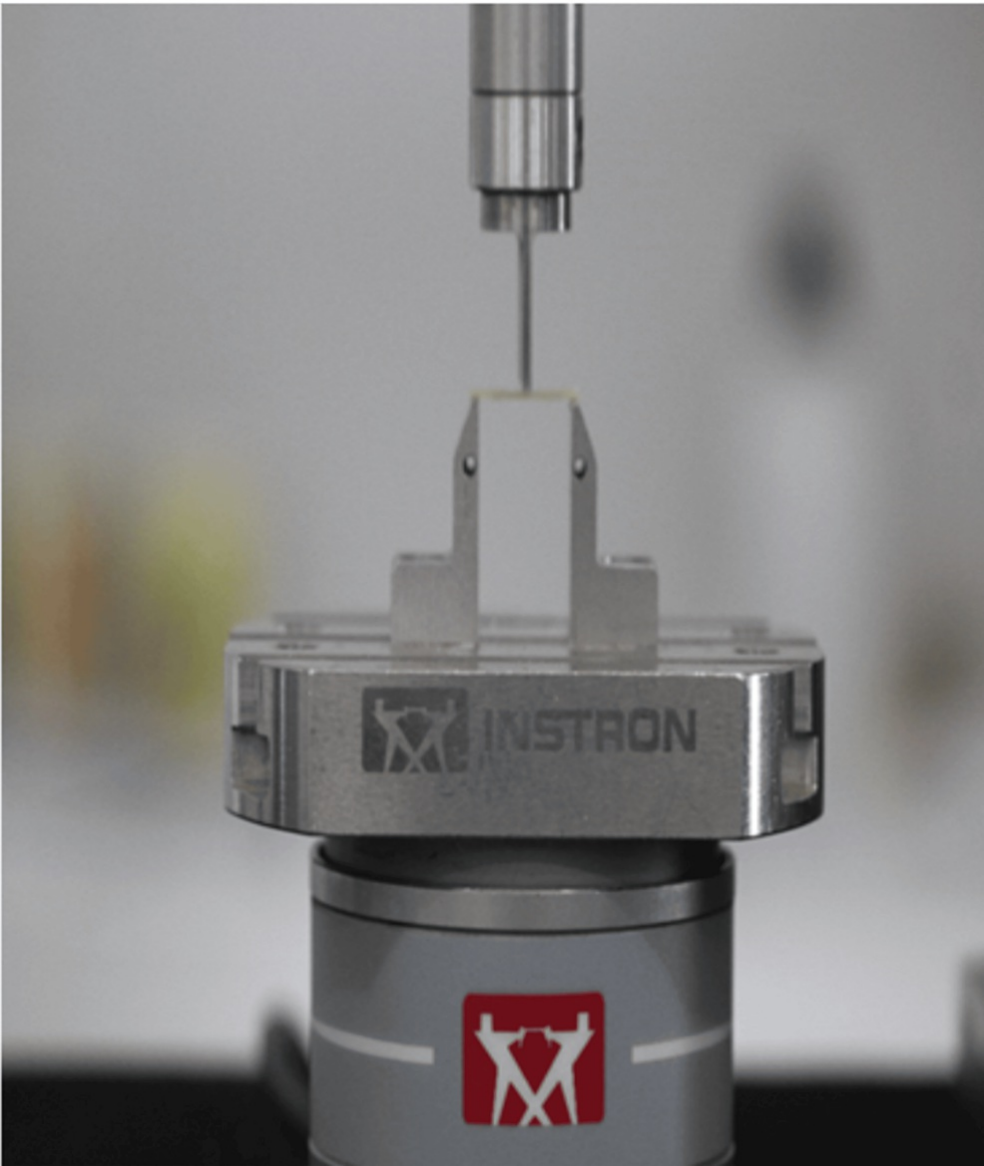
Mounted sample on universal testing machine

The edge chipping test was conducted using an Instron 4501 universal testing machine (Instron Corp., Canton, MA, USA). To maintain precise spacing between the edge and the applied load point, an XY stage was affixed to the testing machine's base. A designated slot with a diameter of 6.35 mm was provided to secure the Vickers indenter (Shanghai Toyo Diamond Tools Co., LTD, Shanghai, China) for applying the load. Additionally, a USB 400× digital microscope was mounted on the setup (Figure 3).

Before initiating the test, several alignment steps were carried out. Initially, the XY stage was set up so that its X and Y movements aligned with the X and Y axes visible in the microscope's image. The sample, affixed to a stainless-steel plate, required its edges to align with the table's Y-axis. This alignment was confirmed by ensuring that the specimen's edge matched up with a designated dashed line. The alignment along the Y-table offset needed to be consistently upheld. After aligning the specimen, the stainless-steel plate was securely fastened to the XY-table. Subsequently, one of the Vickers' diagonals was adjusted to match the stage's Y-direction. To accomplish this, random indentations were made on an aluminum table, and the camera's position was adjusted until the indentation appeared perfectly centered. The test evaluated the force needed to cause a chip in the specimen at a distance of 0.50 mm, a distance commonly considered clinically relevant for edge testing in most studies. The toughness (Kc) was determined using fracture mechanics analysis, which links the critical chipping load (Fc) to the edge distance (d) and the material's toughness (Kc).

$_{Kc}= \frac{_{Fc}}{\beta D^{1.5}}$ 

β = 9.3 is a dimensionless coefficient.

Statistical analysis

Data was collected and tabulated in an Excel sheet (Microsoft® Corp., Redmond, WA, USA). The mean value for edge chipping was analyzed using an unpaired t-test using IBM SPSS Statistics for Windows, Version 26 (Released 2019; IBM Corp., Armonk, NY, USA) after determining the normality of the data set recorded. The statistical significance was set as 0.05.

## Results

**Figure 4:**
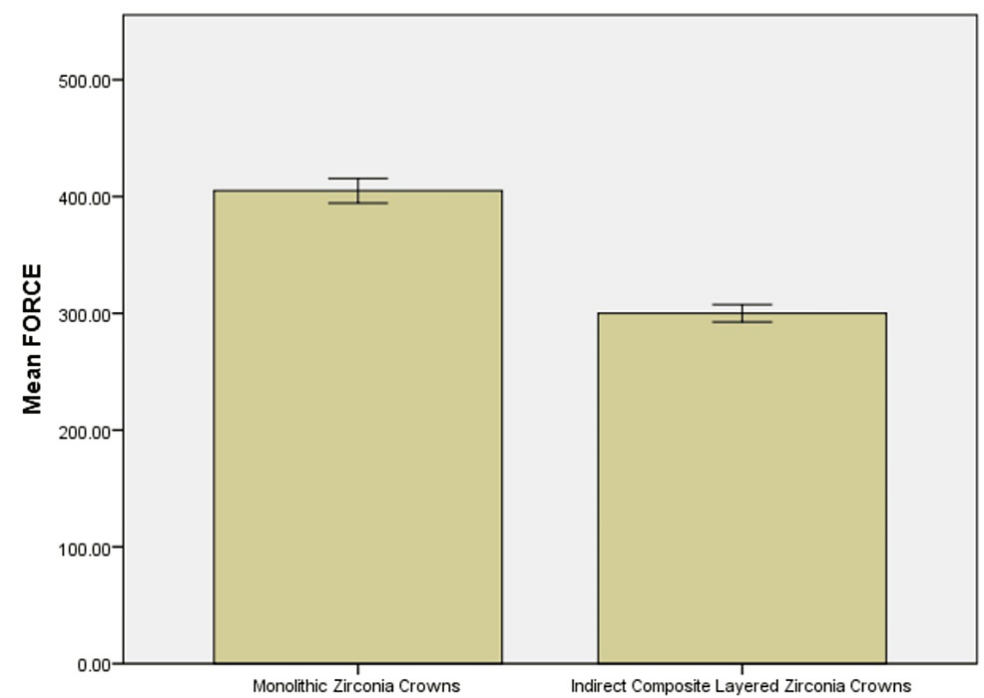
Graphical representation of force required to edge chip

The data presented in the context pertains to the force required to induce an edge chip in two different categories of dental crowns: Monolithic Zirconia Crowns (Group 1) and Indirect Composite Layered Zirconia Crowns (Group 2). The dataset encompasses force measurements for six individual samples within each group. The calculated statistics include the mean force, standard deviation, standard error of the mean, and a 95% confidence interval designed to capture the potential difference between the two groups. Monolithic Zirconia Crowns, identified as Group 1, display a notably higher mean force requirement of 405 N. In contrast, Indirect Composite Layered Zirconia Crowns, belonging to Group 2, exhibit a lower mean force of 300 N. The 95% confidence interval for the difference in mean force provides a range, suggesting that, on average, Monolithic Zirconia Crowns necessitate between 83.43261 N and 109.90072 N more force to induce an edge chip compared to their Indirect Composite Layered counterparts. To elaborate further, the confidence interval indicates a level of certainty regarding the true difference in force requirements between the two crown types. Specifically, the interval of 83.43261 N to 109.90072 N represents the range within which we can reasonably expect the actual difference to lie (Table 1, Figure 4). As the interval does not include zero, it implies statistical significance, suggesting that the observed disparity is unlikely to be a result of random chance. The detailed analysis of the data underscores a substantial and statistically significant difference in the force required to cause an edge chip between Monolithic Zirconia Crowns and Indirect Composite Layered Zirconia Crowns. Monolithic Zirconia Crowns exhibit a lesser susceptibility to edge chipping, necessitating a considerably greater force in comparison to their Indirect Composite Layered Zirconia Crown counterparts.

**Table 1:** Unpaired t-test showing mean force required to edge chip the samples * signifies p-value < 0.05

Groups	N	Mean	Standard deviation	Standard error mean	Significance
Monolithic Zirconia Crowns	12	405.00	16.651	4.806	0.000*
Indirect Composite Layered Zirconia Crowns	12	300.00	11.677	3.371	

**Figure 5:**
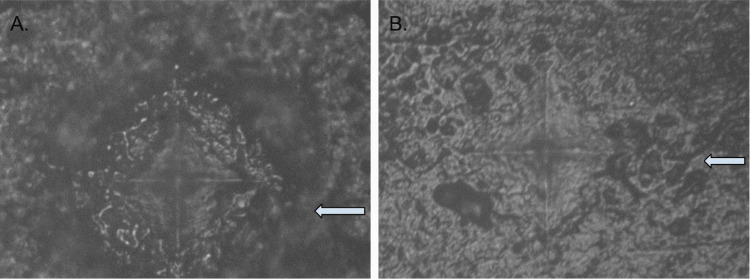
Images magnified at 40x displaying fracture lines in edge-chipped samples A) Monolithic zirconia sample (arrow signifies the indentation made using a Vickers hardness indenter before the entire sample fractured) B) Indirect composite veneer sample (arrow shows propagation of fracture line in indirect composite samples)

Investigations into the failure mechanisms of hand-layered ceramic specimens revealed diverse results, including concurrent breakdown of both the zirconia core and veneer, as well as total material fracture. Microscopically, the fracture line in ceramic samples appeared sharply delineated and well-defined (Figure 5). Conversely, in the indirect composite samples, the fracture line was more diffuse, resembling a drift with gaps between cracks, or exhibiting a broader damaged area.

## Discussion

The primary objective is to understand the real-world performance of dental materials through laboratory tests. However, comparing these studies can be challenging due to differences in research methods and results. Specialized equipment, like the CK 10 developed by Engineering Systems in Nottingham, UK, has gained attention for its effectiveness in assessing chipping resistance in both engineering and dental contexts [11].

However, many studies utilize equipment that incorporates universal testing machines. Regardless of the diverse equipment used, the core principle for evaluating edge chipping remains consistent: applying force with an indenter at set distances from the material's edge and recording the necessary force to cause chipping [12,13]. Chipping often stands out as the leading cause behind the clinical failure of ceramic restorations, explaining why a significant 45% of research attention is directed toward edge chipping in ceramics. Multi-layered ceramic structures, naturally, show greater susceptibility to chipping, regardless of their base material or the detailed fabrication processes, especially when compared to monolithic ceramics, which generally demonstrate better resistance to breaks [14]. Thus, chipping in the veneering ceramic stands out as the primary reason for the failure of multi-layered restorations. Specifically, in rear dental repairs, this type of chipping often appears as a distinctive type of damage from contact, starting from a worn area on the biting surface. Consequently, tiny microcracks that begin below the contact area develop into a single crack within the veneer material [15]. When significant chipping occurs in veneered ceramic setups, these cracks frequently cross the boundary and change direction, moving away from the path of the foundational structure beneath. However, small chips remain in the ceramic veneer, but the underlying structure effectively prevents the spread of these fractures. Due to differences in the thermal and mechanical properties of ceramics that increase the likelihood of chipping, the idea of using monolithic designs has been suggested as a potential solution. These designs naturally show greater resistance to chipping, with some estimates suggesting they can be up to four times more resilient than two-layered versions [16].

Porcelains are complex composites composed of a mixture containing 1% to 30% leucite crystals embedded in a glass matrix rich in oxides [17]. Although often seen as a consistent glass material, dental porcelain's thermal expansion properties are influenced by the leucite particles, causing it to behave differently than typical glasses in a nonlinear manner [18]. The leucite crystal has a coefficient of thermal expansion (CTE) measured at 25 × 10^−6^ °C^−1^ at 450°C. However, this rate decreases to 15 × 10^−6^ °C^−1^ when temperatures go beyond 580°C [19]. At the same time, leucite experiences a reversible shift, known as a displacive or martensitic transformation. Specifically, at normal temperatures, leucite crystals are tetragonal, but they change to a cubic structure at temperatures near 600°C. During the porcelain's cooling phase, the leucite returns to its tetragonal shape from the cubic form, resulting in both structural forms coexisting at approximately 400°C [20]. Significantly, leucite undergoes a marked non-linear change in volume when shifting from its tetragonal to cubic forms. This change occurs at temperatures well below the glass transition temperatures of the porcelain matrix, specifically referred to as TgVM9 at 600°C and TgVM13 at 570°C. Interestingly, this unique volume change of the leucite crystal occurs within the structure of an inherently stiff glass matrix [21,22]. Thus, the inclusion of leucite particles in the glass matrix can create internal stresses, which might accelerate the spread of fractures. On the other hand, indirect composite materials have a more compact structure with a decreased interface, enhancing their ability to withstand mechanical stresses at the edges [23,24]. Furthermore, the cooling phase affects what is known as the CTE, a crucial factor that reduces ceramic durability, a phenomenon not seen in indirect composites [25,26]. The tendency for chipping in indirect composites could be linked to the bonding interactions between the zirconia framework and the applied indirect composite. Based on the results of the study, the null hypothesis remains unsubstantiated.

Despite recognizing the importance of the edge-chipping test for assessing dental materials, the mentioned study has some notable limitations, especially regarding its real-world relevance. These include significant inconsistencies in the experiments, such as differences in the indenter's details, methods for measuring edge distances, classifications of specimens, sizes, surface finishes, speed of loading, and the standards set for recording results. However, future clinical studies and human trials based on the current outcomes could help us establish a concrete clinical protocol, enabling us to provide our patients with better and more predictable outcomes.

## Conclusions

Thermal stresses in porcelain veneers can lead to increased chipping in crowns and fixed dental prostheses (FDPs), even when the base materials are a good match. The design intricacies and the direction of applied forces are known to influence the chipping risk significantly. Since our main goal is to compare materials, we've opted for straightforward specimen shapes to ensure clear evaluations without added complexities. Adding glass to the layered structure enhances the appearance of the zirconia, potentially allowing for better color blending with adjacent teeth by adjusting the glass makeup. While porcelain with glass can be risky in areas with strong biting forces like the back teeth, it might be suitable for front teeth where appearance matters more, given the bite is appropriate for such restorations. In conclusion, when evaluating restorative materials based on both esthetic and functional criteria, monolithic zirconia stands out due to its combination of strength, esthetic potential, biocompatibility, and versatility. While every patient's needs and clinical scenario are unique, monolithic zirconia offers a compelling solution for many restorative challenges in modern dentistry.
